# Acute Myeloid Leukemia: Is It T Time?

**DOI:** 10.3390/cancers13102385

**Published:** 2021-05-14

**Authors:** Meriem Ben Khoud, Tiziano Ingegnere, Bruno Quesnel, Suman Mitra, Carine Brinster

**Affiliations:** 1 CANTHER “CANcer Heterogeneity, Plasticity and Resistance to THERapies”, 1 Place de Verdun, CEDEX, 59045 Lille, France; meriem.ben-khoud@inserm.fr (M.B.K.); tiziano.ingegnere@inserm.fr (T.I.); bruno.quesnel@chru-lille.fr (B.Q.); suman.mitra@inserm.fr (S.M.); 2 Institut Pour la Recherche Sur Le Cancer de Lille (IRCL), 1 Place de Verdun, CEDEX, 59045 Lille, France; 3 Faculté de Médecine, Université de Lille, 59045 Lille, France; 4 Service des Maladies du Sang, CHU de Lille, 59045 Lille, France

**Keywords:** acute myeloid leukemia, T cells, immunotherapy, thymus function

## Abstract

**Simple Summary:**

Acute myeloid leukemia (AML) is a cancer characterized by impaired differentiation and excessive expansion of blood progenitor cells leading to their accumulation in the bone marrow and circulation. The aim of this review is to describe how these leukemic cells can influence the immune system, particularly T lymphocytes that originate from the thymus and are involved in cancers’ and infections’ eradication. We focus on the elderly population, as this disease mainly affects people over 60 years-old. We discuss how AML cells can modify T lymphocytes’ production and functions. We also highlight newly developed therapeutic strategies to improve the anti-leukemic immune response and the clinical outcome of patients.

**Abstract:**

Acute myeloid leukemia (AML) is a heterogeneous disease driven by impaired differentiation of hematopoietic primitive cells toward myeloid lineages (monocytes, granulocytes, red blood cells, platelets), leading to expansion and accumulation of “stem” and/or “progenitor”-like or differentiated leukemic cells in the bone marrow and blood. AML progression alters the bone marrow microenvironment and inhibits hematopoiesis’ proper functioning, causing sustained cytopenia and immunodeficiency. This review describes how the AML microenvironment influences lymphoid lineages, particularly T lymphocytes that originate from the thymus and orchestrate adaptive immune response. We focus on the elderly population, which is mainly affected by this pathology. We discuss how a permissive AML microenvironment can alter and even worsen the thymic function, T cells’ peripheral homeostasis, phenotype, and functions. Based on the recent findings on the mechanisms supporting that AML induces quantitative and qualitative changes in T cells, we suggest and summarize current immunotherapeutic strategies and challenges to overcome these anomalies to improve the anti-leukemic immune response and the clinical outcome of patients.

## 1. Introduction

Acute myeloid leukemia (AML) is a clonal disorder characterized by the differentiation blockade and rapid proliferation of myeloid primitive cells (hematopoietic stem cells (HSC), lymphoid/myeloid progenitors and myeloid precursors) due to the transformation of regular hematopoiesis programs [1]. It represents the most common acute leukemia in the adult population, and its incidence rises with age, with ~3 to 4 cases per 100,000 persons per year worldwide. The median age at diagnosis of AML is estimated over 60 years old. The accumulation of undifferentiated myeloid cells characterizes the disease, termed blasts, in the bone marrow (BM) (>20%) and peripheral blood of patients leading to altered hematopoiesis associated with anemia, thrombocytopenia, neutropenia, and immunodeficiency. Depending on the affected myeloid lineage (monocytes, red blood cells, platelets, and granulocytes) and its maturation stage, AML was first classified into cytomorphological subtypes (myeloblastic, promyelocytic, erythrocytic, myelomonocytic, monoblastic, monocytic, and megakaryocytic). However, a molecular classification has been adopted according to the cytogenetic changes found in leukemic blasts. Some aberrant karyotypes with translocations, duplications, or deletions of chromosomes were found, and different patterns of mutations affecting genes involved in the hematopoietic cell proliferation and differentiation. Although 50% of the patients present a normal karyotype, the combination of mutations associated with an aberrant karyotype can be complex. Such AML patients’ clusters can be categorized into favorable, intermediate, and unfavorable treatment prognosis groups. Overall, different types of AML exist and the World Health Organization (WHO) has classified them into 6 groups: with recurrent genetic abnormalities, with myelodysplastic syndrome-related features, therapy related, not otherwise specified, myeloid sarcoma-associated and myeloid proliferations related to Down syndrome.

Frontline treatment for AML in adults mainly includes chemotherapy agents (notably cytarabine and anthracyclines–doxorubicin and daunorubicin) or allogeneic hematopoietic stem cells transplantation (allo-HSCT). Intensive chemotherapy induction and consolidation phases lead to 50 to 80% complete remission (RC) (<5% blasts in the BM) in patients younger than 60 years old. Clinical responses in patients older than 60 years are more unfavorable with poor overall survival. When present, the cytogenetic abnormalities (mutations and, or gene fusions) can help to follow-up and detect residual leukemic cells known as Minimal/Measurable Residual Disease (MRD) after treatment. However, this latter is not always predictive of the disease evolution, and relapses can occur in 50% of cases between 2- and 48-months post-chemotherapy. Allo-HSCT is the alternative treatment for relapsed and refractory AML. However, high-risk mortality (~30%) due to the development of acute Graft-versus-Host Disease (aGvHD) in transplanted recipients further complicates its use. Thus, considering the 5 year-overall survival rates for AML-affected patients (~30%), there is an urgent need to develop new therapeutic strategies.

The recent discovery of molecular and cellular mechanisms leading cancer cells to escape immune surveillance has opened the way to immunotherapy. This review will focus on cell lymphopoiesis and peripheral response during AML and aging as this pathology primarily affects the elderly population. Here, we discuss how leukemic cells can worsen the age-associated thymic involution and peripheral T-cell senescence and we summarize the current strategy and challenges in developing immunotherapy-based approaches for treating patients with AML.

## 2. Thymic Involution Due to Aging in Healthy Persons

The thymus is an organ composed of two lobes subdivided in lobules by septa of connective tissue emerging from the surrounding capsule. It constitutes the primary lymphoid organ for T cell development and generates new naive antigen-specific T cells and their output into the periphery. The majority of circulating T lymphocytes (90–95%) harbor a TCR composed of α and β chains (αβ^+^ TCR); γδ^+^ TCR T cells are also present but to a minor extent. In adults, thymopoiesis begins with the entry of bone marrow lymphoid-committed progenitors identified as early T-lineage progenitors (ETP) in the double negative (DN) CD4^−^CD8^−^ thymocytes. BM cells that seem to be competent for this process are lymphoid-primed multipotent progenitors (LMPP) and common lymphoid progenitors (CLP), although their respective in vivo contribution remains to be determined. Once in the thymus, these cells undergo maturation stages from DN1 to DN4. These DN4 cells begin to differentiate into double-positive (DP) CD4^+^CD8^+^ cells and then, single-positive (SP) CD4^+^ or CD8^+^ mature naive T lymphocytes after their T cell receptor (TCR) rearrangement and the processes of positive and negative selections. These latter are respectively mediated by interactions with the cortical and medullary thymic epithelial cells (TECs) which provide self-antigen presentation through CMH/HLA class I/II complexes for the deletion of auto-reactive cells. Thymus tissue reaches its maximum size within the first 12 months of life, then starts to decline as early as the second year of life at a rate of approximatively 3% per year until middle age (40 years old), and then 1% per year [2]. In contrast to mice, where the total thymic size is reduced in aging individuals, in humans, it remains constant, but the thymic tissue responsible for the generation of T lymphocytes is gradually replaced by connective and adipose tissues [2,3]. However, although old individuals’ thymus organization is altered, it maintains a constant thymopoiesis. It allows lifespan a permanent export of naive T cells that express a diverse antigen receptor repertoire [4,5,6]. Accordingly, this phenomenon was demonstrated by direct visualization and functional studies of thymocytes isolated from adult thymic tissue [5,6]. The development of assays quantifying TCR excision circles (signal joint (sj)TREC and their ratio to βTREC), episomal fragments produced during the TCR gene rearrangements, also allowed the detection of cells originating from the thymus and their dynamics in the periphery [7]. These sequences are unique to naive αβ^+^ T cells but are diluted following cell proliferation. Their frequencies decline with age about ten times greater than the naive subset in CD4^+^ and CD8^+^ T cells [8,9,10]. As best examples, it was shown that centenarians present 2 to 5-fold less naive (CD45RA^+^CCR7^+^) cells in CD4^+^ and CD8^+^ T populations, respectively, compared to young subjects and sjTREC can still be detected to a much lesser extent (about 10-fold) [10,11,12], revealing a functional thymic output.

### 2.1. Age-Associated Factors Affecting Thymocytes Numbers and Function

Age-dependent defects leading to thymic involution are not fully understood, but involve both the developing thymocytes and stromal cells’ inter-dependency.

Hematopoietic stem cells (HSCs) are responsible for producing CLP in the BM, which will then migrate to the thymus. In humans and mice, the proportion of HSCs increases in the BM with age, accompanied by intrinsic defects in gene expression profiles leading to different sub-populations, including lymphoid- (Ly-HSC) and myeloid-biased HSC (My-HSC). In the elderly, a more pronounced increase in My-HSC numbers and higher levels of differentiated myeloid cells were observed in BM and blood [13,14]. Studies performed in aged mice have demonstrated that BM-derived CLP were unlikely to be affected in their migration to the thymus but sorted intra-thymic ETP (enriched in the DN1 fraction) presented enhanced sensitivity apoptosis and reduced proliferation, particularly though the increased Ink4a expression [15]. These intrinsic defects led to lower numbers of subsequent maturation stages of DN2 and DN3 subsets [16,17]. Diminished CD3 expression on DP and SP thymocytes was also found in aged mice compared with young mice, resulting in an altered response to mitogen stimuli [18]. Additional studies showed that old lethally irradiated mice reconstituted with T-depleted bone marrow cells from young counterparts were still able, even to a lesser extent (about 50%), to produce a normal T-cell progeny. Thus, the thymic stroma demonstrated a functional capacity to generate mature T lymphocytes. However, its size and composition were affected by age (more pronounced reduction in the cortex than medulla area) [19].

### 2.2. Histological, Cellular, and Transcriptional Changes in the Thymic Stromal Cells with Age

Apart from thymocytes, the thymic stromal microenvironment is composed of fibroblasts, endothelial, mesenchymal (stem cells or pericytes), neural, epithelial cells (TECs), B cells and medullary macrophages/dendritic cells. The endothelial cells form the post-capillary venules (PCV) in the cortico-medullary junction that allow the import of thymic progenitors from the BM and the export of mature T lymphocytes through the expression of adhesion molecules. The thymic fibroblasts synthesize growth factors that are essential for thymocyte survival and proliferation (Stem cell factor- SCF), TECs (fibroblast growth factors- FGF7 and 10) and endothelium (vascular endothelial growth factor-V-EGF) maintenance. The TECs synthesize and secrete different factors that control thymocytes survival, proliferation and differentiation. They produce cytokines (notably interleukin-7- IL-7), growth factors (G-CSF, GM-CSF, insulin-like growth factor 1 (IGF-1)), hormones (thymopoietin, thymulin, thymosin, thymic humoral factor (THF), the growth hormone (GH)) and glucocorticoids (GC). Medullary TECs and macrophages/dendritic cells express and present self-antigens (from different tissues) to the ongoing differentiating thymocytes.

In rodents, thymic atrophy begins as early as 7 weeks of age. Disruption in the thymic stroma structure is obvious by 3 months of age, with changes in the cortico-medullary junction and a more pronounced reduction in the cortical than medullary zone. These observations precede the hematopoietic changes (intrinsic defects in HSC and ETP), which can be detected at around 7 months. Thus, the stromal population seems to be a primary target for age-associated defects. Transcriptional gene profiling studies assessed in the different stromal cell types between 1 and 6 months of age revealed different signatures. Decreased expression of cell cycle-associated genes was detected for TECs, and pro-inflammatory genes increase for thymic dendritic cells. The aging process also alters the expression of growth factors by TECs and fibroblasts [20]. Venables and colleagues showed, in recent results, that using fluorescent confocal microscopy, cortical TECs (cTECs) could undergo dynamic size changes with cell projections retraction during thymic atrophy in aged mice (12 months-old), leading to the reduced cortical area. Total TECs numbers and medullary TECs (mTECs) morphology were found unchanged during thymic atrophy [21]. In contrast, Griffith et al. demonstrated the loss of mTECs network and of the tissue-restricted antigens’ expression required for self-reactive thymocyte selections [22].

With age, different types of unilocular and multilocular lipid-laden cells accumulate within the capsule and septa regions as well as in the perivascular space of PCV [23,24]. These cells produce pro-inflammatory molecules such as leukemia inhibitory factor (LIF), tumor necrosis factor-α (TNF-α), IL-1, IL-6 and other family members [25]. Their presence could also lead to lipotoxic “danger associated molecular patterns” as revealed by the evaluation of lipidomics profiles of the whole thymus between young (4 months-old), middle-aged (12 months-old), and old (18 months-old) mice [26]. Indeed, high levels of intra-thymic ceramides and cholesterol were co-localized with macrophages in the medullary area [26,27]. These molecules were shown to be responsible for the activation of caspase-1 and the Nlrp3 inflammasome, leading to pro-inflammatory cytokine production (TNF-α, IL-1β, IL-18, IL-6). Fibroblasts and TECs were shown to all express the IL-1R1. In contrast, mTECs define the IL1 antagonists (IL-1R2 and IL-1RA), confirming the damaging action of IL-1 mainly on cTECs [23,27]. Thus, these studies could explain the unbalanced decline of the cortical over the medullary area with age.

## 3. Thymus Function in AML-Affected Patients

The thymic size and histology have been poorly evaluated in AML newly diagnosed patients. Lower numbers of sjTREC in peripheral T cells were observed in AML-affected patients compared to age-matched healthy individuals, suggesting a dysfunctional function [28]. However, experimental animal models and organoids will help understand these features better. As AML blasts invade BM and blood, one can speculate that thymic function could be affected as far as lymphoid T-cell progenitors migrate from the BM to the thymus.

## 4. Leukemic Cells and Medullary Regular Hematopoietic HSC/T-Cell Progenitors’ Interactions

**Figure 1:**
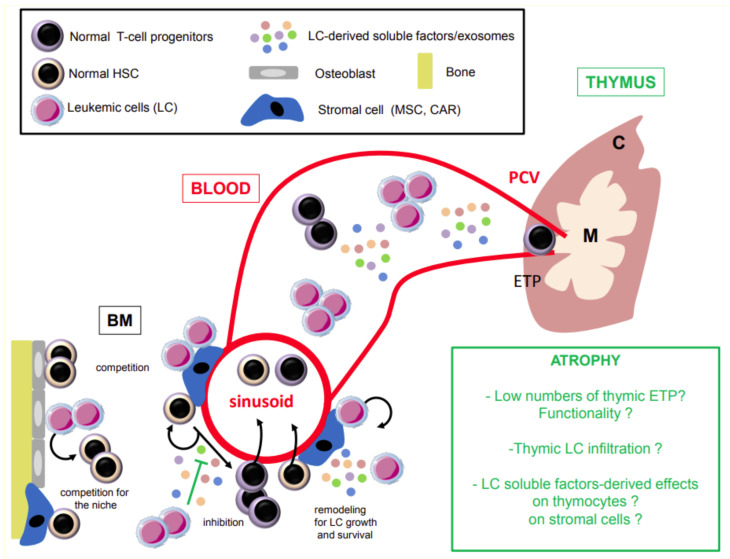
Potential mechanisms leading to thymic atrophy during AML. In the bone marrow (BM), leukemic cells secrete soluble factors that can directly inhibit HSC proliferation and differentiation. They can also produce exosomes that modify the stromal and endothelial cells expression profiles and promote leukemic cells growth and survival. Ultimately, they also compete for spaces and niches of the normal HSC which are required for their survival and proliferation. The alteration in HSC proliferation and differentiation leads to reduced numbers of T-cell progenitors migrating and entering the thymus (ETP) though the blood stream. High concentrations of soluble factors produced by leukemic cells in the blood can also alter the thymic cells (thymocytes and stromal cells) functions and differentiation. HSC: hematopoietic stem cells; CAR: CXCL12-abundant reticular cells; MSC: mesenchymal stem cells; ETP: early T-cell lineage progenitors; BM: bone marrow; PCV: post-capillary venules.

Hematopoiesis in the BM is dependent on specialized niches (endosteal and peri-vascular regions) that home and regulate the HSC and progenitors’ proliferation and differentiation capacities. Clonal leukemia-initiating cells are now known to derive either from transformed normal HSC and/or downstream progenitors. These latter include myeloid and/or LMPP or more committed progenitors like GMP (Granulocyte Monocyte Progenitors), depending on the AML subtype and its associated genetic abnormalities [29,30,31]. When cells are affected by the leukemogenic process (mutations, fusion genes), they co-exist with their regular counterparts and compete for space and niches [29,32]. They were shown to impair normal HSC proliferation and differentiation through direct secretion of inhibitory molecules and remodel the BM microenvironment to favor their survival and growth [33,34] (Figure 1). Primary AML blasts were found to produce large amounts of diverse cytokines (including GM-CSF, G-CSF, IL-3, IL-6, IL-1α, β, TNF-α, and SCF) and chemokines (CCL2/3/4/5/13/17/22/24, CXCL1, CXCL2, CXCL5, CXCL8, and CXCL9 to 11) [35]. Sera and BM levels of these molecules can vary individually in AML-diagnosed patients without any correlation with age (< or > 65 years old), AML subtype, and associated genetic anomalies. Using AML patient blasts-derived xenograft mouse models, the leukemic cells were shown to produce elevated levels of CCL3 in the BM and to suppress the normal HSC differentiation into erythrocytes [36]. BM stromal and endothelial cells were recently found to internalize exosomes released by AML blasts. They led to the down regulation of expressed factors mandatory for the normal HSC homing (like CXCL12), thus sequestering them away from their niche and disrupting their function [37]. These exosomes also favor an adipogenic rather than osteogenic and chondrogenic differentiation from mesenchymal stromal progenitors.

## 5. Leukemic Cells and Thymic Cells Interactions

Whether leukemic cells could infiltrate the thymus of AML-affected patients and release these molecules in situ (with or without the help of exosomes) remains to be elucidated. However, significant chemokine receptors (CCR7 and CCR9) involved in the migration of immune cells to the thymus were not shown to be expressed by AML blasts; neither was the secretion of their respective corresponding chemokines (CCL19/21/25). Tan and co-workers found that AML cells could produce significant amounts of sphingosine-1-phosphate (S1P) in vitro for their survival and proliferation. Although their blood levels were found lower in AML-affected patients than in healthy donors, more elevated levels of sphingosine, sphinganine and ceramides were respectively detected [38,39]. Thus, whether these lipidic molecules could also contribute to the thymic involution could be questioned. Similarly, AML blasts can produce different cytokines (SCF, pro-inflammatory) that could potentially affect the survival, phenotypes, transcriptional gene profiles and secretomes of stromal cells, thymocytes and even lipid-laden multilocular cells.

## 6. Thymic Features in AML-Bearing Mice

Using young mice (6 weeks-old) and an immune-competent AML experimental mouse model, we recently showed an accelerated and premature involution of the thymus during leukemic development [40]. This thymic atrophy was characterized by reduced numbers of thymocyte subsets (notably DP) and peripheral T lymphopenia with increased levels of activated/memory cells [40]. Leukemic cells infiltrated the bone marrow, blood, lungs, liver, and spleen, but very few cells (<0.25%) were found in AML-bearing mice’ thymus. High levels of intra-thymic and systemic (sera) CCL2 chemokine were detected during AML and were found to be associated with this involution independently of myeloid cells recruitment [40]. Although HSC proportions in the bone marrow were not evaluated at that time, we also observed significantly decreased progenitor cells of B-cell, monocyte and granulocyte lineages during AML [40,41]. These characteristics were not observed in the spleen, another critical site of hematopoiesis in mice. Splenomegaly was instead due to increased numbers of differentiated immune cells rather than AML blasts infiltrates [41]. Preliminary and unpublished additional data from our team also reveal reduced numbers of DN subsets in the thymus of young leukemic mice compared to age-matched control animals (unpublished data, personal communication). Similar features have already been described in other cancers. Further analyses are continuing for this AML-induced thymic atrophy to provide more insights on the specific mechanisms involved and their relevance for AML-affected patients.

## 7. Peripheral T-Cells in Healthy Aged Persons

In the elderly, the overall peripheral T-cell compartment is maintained. Still, its composition shows a remarkable shift from naive to memory T cells, particularly in the CD8^+^ subset, when compared to young individuals. Naive T cells are antigen-inexperienced, express the molecular surface markers CD45RA, CD62L, CD27, CD28, CCR7, CD127, and can produce interleukin-2 (IL-2) and display unique proliferative response upon stimulation. Their homeostasis is maintained by two mechanisms: their emigration from the thymus and the expansion of the peripheral pre-existing naive population. This clonal expansion was shown to be maintained in the periphery by tonic signaling (through MHC/HLA class I or II/self-antigens complexes) and cytokines. Both populations can be distinguished with the help of the CD31 marker as naive CD31^+^ T cells were shown to contain higher amounts of sjTREC than CD31^−^ counterparts and enriched in recent thymic emigrants (RTE) [8,42]. Den Braber and colleagues demonstrated that the average rate of naive T cells originating from the thymus throughout adulthood was estimated ~11% of the naive peripheral T cell population [11]. The analysis of the naive TCR repertoire in both young (20–35 years old) and aged (70–85 years old) persons indicated a significant decline in TCR richness and higher clonality with age (particularly after 70 years old) [43,44]. Among the CD4^+^ T population, the peripherally expanded cells (CD31^−^) also exhibited a more restricted repertoire (oligoclonal) than RTE (CD31^+^) in young and old adults, revealing the essential role of the thymus in the generation of new TCR specificities [45]. Recently, Egorov et colleagues noticed changes in the Vβ chain CDR3 region (decreased length and reduced insertions of random nucleotides) with aging leading to a higher affinity to self-antigens [43]. Although still controversial, these intrinsic modifications could explain the holes (absence of certain TCR chains) observed in the repertoire with aging and the better fitness of certain clonotypes to tonic signaling in the periphery and their selective expansion [43,44].

Short-lived effector (T_EFF_) and memory (T_M_) T cells are generated from naive cells following the antigens’ initial encounters. T_EFF_ can produce various cytokines depending on their polarization, which clear the antigens. T_M_ respond rapidly upon antigens re-exposure and can be divided into different subsets (central memory, effector memory, stem cell memory, resident memory…). With age, antigen-specific end-differentiated effector memory T cells accumulate at the expense of normal T_EFF_, particularly in the CD8^+^ compartment. Their proportion rises particularly during latent chronic viral infections such as cytomegalovirus. These cells present shortened telomeres, restrained TCR repertoire, lose the expression of CD28, CD27, regain the expression of CD45RA and upregulate CD57, KLRG-1 and TIGIT. They present a reduced proliferative capacity but enhanced cytotoxicity by secreting granzymes/perforins and pro-inflammatory cytokines and are characterized as senescent T cells. The naive T-cell compartment can also comprise senescent cells due to their constant homeostatic proliferation with age.

## 8. Peripheral T-Cell Phenotypes and Functions in AML-Affected Patients

AML-affected patients can present lymphopenia in the periphery or reduced T lymphocytes with a predominant proportion of effector/memory over naive cells. The analysis of sjTREC contents in peripheral T cells has described lower numbers of sjTREC than age-matched healthy individuals [28], suggesting a diminished thymic output during AML. However, further studies (notably on CD31 subsets) are still missing to provide more precise insights on the naive T-cell pool kinetics. When measuring TCR repertoire diversity, research groups have demonstrated oligoclonal and skewed αβ^+^T-cell repertoires with holes and overrepresentation of some Vβ clonotypes in AML-affected patients, whatever their age [46,47]. Similar results were found by Jin and co-workers when exploring the γδ^+^ T-cell repertoires [48]. These findings suggest (1) an increased deletion of leukemia-specific T-cell clones in the thymus leading to tolerance and/or (2) the preferential expansion of some TCR subfamilies through leukemia-associated antigen(s) (LAA) stimulation in the periphery. Some HLA class I- and II-restricted epitopes from LAA have been identified, but understanding of their clinical specific T-cell responses in patients are still limited [49,50]. It also remains unknown whether these circulating LAA and their derived peptides can be adequately presented by thymic epithelial cells for the thymus positive and negative selections during AML. Whether autoreactive T-cell clones could also expand through epitope spreading in this inflammatory- and lymphopenic-induced AML environment remains to be determined. In addition to these observations, peripheral CD4^+^ and CD8^+^ T cells exhibit various dysfunctional properties. Alterations in their TCR signaling, in their phenotype, in their gene expression profiles, and their inability to form synapses with AML blasts have been described [51,52]. Leukemic cells are associated to localized or systemic immunosuppressive effects on T cells. Accordingly, T cells from AML patients demonstrate features of T cell exhaustion and senescence. 

### 8.1. Exhausted T Cells (T_EX_)

Cytotoxic CD8^+^ T cells (CTLs) are the main effectors for eliminating leukemic cells. They produce different cytokines such as IL-2, interferon-γ (IFN-γ), TNF-α and cytolytic granules (granzymes and perforins). CD8^+^ T_EX_ cells present elevated expression of PD-1 (CD279) and other co-inhibitory receptors (IR) such as: CTLA-4 (cytotoxic T lymphocyte antigen-4/CD152), Lag-3 (lymphocyte-activation gene 3), Tim-3 (T cell immunoglobulin domain 3), CD244, CD160, KLRG-1 (killer cell lectin-like receptor subfamily G) and TIGIT (T cell immunoreceptor with Ig and ITIM domain) [53,54,55]. They can co-express one or all of the IR depending on their exhaustion severity. They exhibit reduced proliferation, cytotoxic activity and impaired cytokine production. They typically lose their IL-2 production first, followed by the TNF-α output and IFN-γ, according to their exhaustion state [55,56]. T_EX_ also present a higher sensitivity to death with upregulation of apoptotic genes such as *CASP1* and *FASLG*. Other studies have demonstrated the role of transcription factors in T-cell exhaustion including Eomes and Tbet. CD8^+^ T_EX_ differentially express Eomes and Tbet (Eomes^+^ Tbet^lo^) during AML [57]. The accumulation of such CD8^+^ T_EX_ in patients at diagnosis or after allo-HSCT was shown to be predictive of their resistance to chemotherapy treatment or relapse, respectively [58,59].

### 8.2. Other Effects of Leukemic Blasts on T Cells’ Proliferation, Function and Survival

Additional suppressive mechanisms of human AML blasts on peripheral T cells can affect their activation, proliferation (leading to anergy) and survival. Among them, the high expressions of the indoleamine 2,3-dioxygenase (IDO) and arginase, two enzymes released by leukemic cells in the PB favor tryptophan and arginine depletions, respectively [60,61]. Kynurenines production after tryptophan catabolism by IDO is associated with the inhibition of proliferation (or anergy) or apoptosis of surrounding T cells. Similarly, the secretion of arginase 2 deprives T cells from arginine required for their proliferation [60]. AML blasts and CD8^+^ T cells compete for glutamine uptake in the microenvironment as this amino acid is critical for leukemic cells’ survival and cytolytic function of CD8^+^ lymphocytes, respectively. Thus, as leukemic cells grow, they deprive T cells of their needed glutamine, impairing their anti-tumor response [62,63,64].

Soluble Tim-3 and Gal-9 molecules released by AML blasts inhibit CD8^+^ T-cell expansion [65] as well as interactions (notably though VISTA molecules) with myeloid-derived suppressor cells (MDSC) which increase in the PB during AML [66,67]. Reactive oxygen and nitrogen species (ROS) released by leukemic blasts and MDSC are also responsible for inhibition of T cells’ proliferation through the chemical alteration of the TCR or IL-2 receptor signaling.

### 8.3. Role of Regulatory T Cells

Regulatory T cells (Tregs), a CD4^+^ T-cell subset, are critical for maintaining peripheral homeostasis and tolerance against self-antigens and suppressing over reactive harmful immune responses. Yet, they can also suppress anti-tumor specific T-cell responses. They can originate either from the thymus (natural Tregs- nTregs) or be induced from naive CD4^+^ T cells in the periphery (inducible Tregs- iTregs). nTregs mediate their suppressive activity via diverse cell contact-dependent or -independent mechanisms, iTregs through the production of TGF-β and/or IL-10 [68]. Different studies have shown increased Treg frequencies in BM and blood of AML patients at diagnosis compared to healthy volunteers [69,70]. Their association to poor prognosis at diagnosis is still controversial but they were shown to persist after intensive chemotherapy and could be more predictive of relapses [71,72]. Such increased frequencies of peripheral (splenic) Tregs were also observed in our experimental AML-bearing mouse model [40]. nTregs derived from AML patients present an enhanced suppressive activity compared to healthy volunteers. AML-associated nTregs express high levels of both immunosuppressive ATP ecto-nucleotidase CD39 and cAMP that ultimately inhibit conventional T cells proliferation [73]. Elevated levels of TGF-β, IL-10 and IL-35 were also detected in the peripheral blood plasma of AML patients compared to healthy donors. IL-35 was found to be produced by nTregs and shown to inhibit effector T cell proliferation while promoting nTregs and AML blasts expansions [74]. IDO produced by leukemic cells can generate iTregs in vitro and was expressed by mesenchymal stem cells (MSC) derived from AML patients [61,75]. Similarly, PD-L1^+^ or ICOSL^+^ AML blasts could generate iTregs and could also favor the proliferation of PD1^+^ or ICOS^+^ nTregs [61,76]. Finally, Wang and collaborators recently highlighted the role of the TNF-α/TNFR2 signaling pathway in the in vitro expansion of AML-derived nTregs [77]. Conjointly, TNF-α could also contribute to the ICOSL molecule up-regulation on AML blasts [76]. nTregs could also induce senescent CD4^+^ or CD8^+^ T cells (CD27^low^ CD28^low^ SA-β-gal^+^) in vitro and in vivo. These latter exhibit a suppressive activity (iTregs) but also produce significant amounts of pro-inflammatory cytokines (IFN-γ and TNF-α) [78]. This phenotype could be reversed in the presence of TLR8 ligands and MAPK signaling inhibitors [78].

### 8.4. Senescent T Cells

Different groups have likely shown an increased percentage of CD8^+^ T cells with a senescent phenotype (CD28^−^CD57^+^INFγ^high^ TNFα^high^) in AML-affected patients compared to age-matched healthy donors. Such increase was observed in refractory patients or at relapse after the chemotherapy treatment (standard induction regimen) [79,80]. Whether these senescent T cells are LAA-specific and naturally derived or induced by nTregs during AML remains to be elucidated. However, their generation’s mechanism would provide insights into their possible functional reversion by immunotherapy.

**Figure 2:**
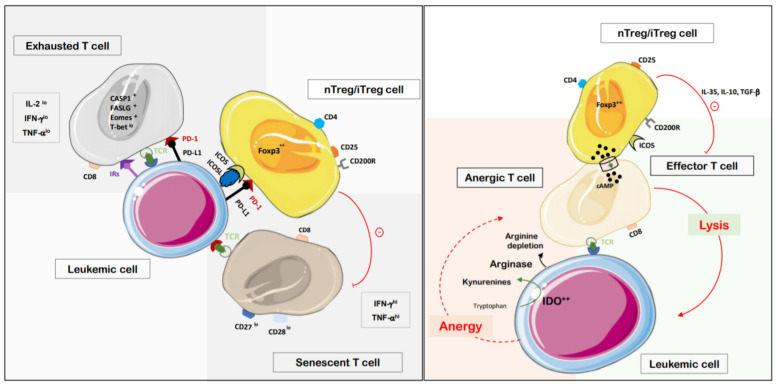
Principal mechanisms leading to peripheral T-cell dysfunctions during AML. In periphery or BM, T cells can present exhausted, anergic and senescent phenotypes with dysregulated functional activities (reduced levels of proliferation, cytotoxicity and cytokines production). Leukemic blasts are mainly responsible for these defects through persistent antigen presentation, inappropriate co-stimulatory signaling and induction of Tregs (ICOSL, PD-L1). Natural Tregs are also recruited abundantly and can participate in these processes. nTreg cell: natural regulatory T cell; iTreg cell: induced regulatory T cell; IL: interleukin.

Thus, AML blasts exert T-cell subversion through multiple mechanisms (Figure 2), but a recent longitudinal study underlined the restoration of these CD8^+^ T-cell dysfunctions after chemotherapy in responder patients [79]. These findings are now opening the way to new immunotherapeutic strategies that could inhibit reversible leukemic cells deleterious effects on immune T-cell response. Such treatments would also offer less toxicity, convenient infusion and better tolerability in the elderly.

## 9. Strategies to Boost the T-Cell Immunity in Old AML-Affected Patients

### 9.1. Rejuvenating the Thymic Function?

The thymic function only modestly contributes to the naive T-cell compartment content (estimation of ~11%) in the elderly but remains essential for its repertoire diversity (polyclonality). AML-affected patients present an altered thymic output as revealed by reduced sjTRECs numbers and the exact mechanisms involved in this impairment must be determined. This thymic output becomes even more important in cases of lympho-depletions that occur after treatment with chemotherapeutic agents and allo-HSCT. Intensive chemotherapy induction treatment is followed by early CD4^+^ and CD8^+^ T lymphocyte recovery within the first month [72]. It was shown that some residual peripheral T cells persisted through chemotherapy and underwent homeostatic expansion. However, they included high frequencies of nTregs (due to their slow proliferation), and their TCR repertoire was found oligoclonal [72]. As chemotherapeutic agents are known to damage the thymus structure on both maturing thymocytes and TECs (particularly mTECs) and delay the thymic output, it remains unknown if this latter could participate in the T lymphocyte compartment recovery and long-term remission [81,82]. Accordingly, different recent studies suggest that maintaining a practical thymic function in the elderly population could lead to better survival [83,84]. Thus, therapies aiming at improving the thymic T-cell production would be promising in preventing or treating age-related diseases. Strategies aimed at boosting the thymic function have encountered, so far, satisfactory results in preclinical models and clinical trials, even if transient [85]. Indeed, the thymic size and structure of the involuted thymus can momentarily be restored by administering growth factors or hormones (GH, IGF-1, FGF7), usually expressed either by stromal cells or differentiating thymocytes for their survival, differentiation, and proliferation. Their expression decreases with age, and they have been considered a treatment to counteract age-associated thymic involution and its effects. However, such an approach also has systemic effects, and the proliferation of AML blasts has to be considered, as IGF-1 was shown to favor their expansion in vitro [86]. The recent discovery of TEC progenitor cells (TEPC) residing in the thymus and their in vitro generation from induced pluripotent stem cells have opened the way to new therapeutic strategies to re-establish permanent thymopoiesis [87]. Normal HSCs and their niches are thought to be preserved (even in low numbers) during AML, and successful elimination of leukemic cells can restore their functions. A limiting point resides in the age of these HSC in the elderly and their intrinsic defects (myeloid-bias development of the progenitors, transcriptional changes…). However, Haynes et al. found that CD4^+^ T cells generated from old HSCs in the young thymic microenvironment were functional [88]. One could then speculate about the infusion of such autologous TEPC in order to regenerate the thymic TEC compartment and its inter-connection with maturing thymocytes. Time considerations and doses and accurate antigen presentation for self-tolerance need to be taken into account, but these investigations offer great promise for improving T-cell response.

### 9.2. Improving Anti-Leukemic T-Cell Immunity in the Periphery?

**Figure 3:**
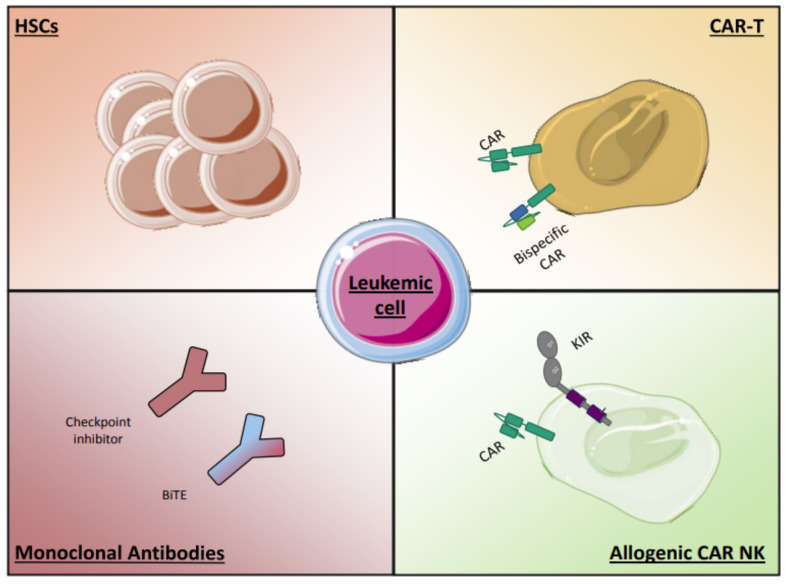
Promising immunotherapies for the treatment of AML. New immunotherapeutic strategies are under development for the treatment of AML: monoclonal and bi/trispecific antibodies (inhibition of immune checkpoints, targeting of leukemic cells), CAR-T or –allogeneic NK cells against AML blasts surface markers, and improvements in allo-HSCT.

Several immunotherapeutic drugs have been investigated alongside chemotherapy in recent years. Here we summarize some of the most promising immunotherapies in AML (Figure 3).

## 10. Immune Checkpoints Inhibitors

Immune checkpoints regulate self-tolerance and protect tissues from damage under normal physiological conditions. It is now well known that tumors exploit these mechanisms to escape immune surveillance [89]. Because many of the immune checkpoints are triggered by ligand-receptor interactions, they can be blocked by antibodies (mAb) or modulated by soluble forms of ligands or receptors. It is now clear that immune evasion mechanisms are active in patients with AML. Moreover, T-cells express various immune checkpoints in AML [90], paving a rationale for immune checkpoint therapies in this disease.

The checkpoints with active ongoing trials using mAb in AML are CTLA-4, PD-1, TIM-3, and TIGIT.

CTLA-4 is the first discovered immune checkpoint. It is a competitive agonist of CD28/B7 costimulatory interaction [91,92]; it thus acts as an inhibitor of T cell effector function. Ipilimumab is a mAb targeting CTLA-4 with active clinical trials in AML (NCT02530463, NCT01757639). The programmed cell death 1 (PD-1)/PD-L1 axis plays a significant role in immune evasion and T-cell exhaustion in AML [93,94,95,96]. Mainly found in CD8^+^ T cells, PD-1 prevents the tumors’ active killing [96,97]. The blockade of PD-1/PD-L1 interaction restored T cells function in a mouse model of AML [95]. Trials with different antibodies block the PD-1/PD-L1 axis: Pidilizumab [98], Nivolumab [99], pembrolizumab, durvalumab, and atezolizumab (reviewed in [90]) are currently in progress to determine efficacy. TIM-3 is a co-inhibitory receptor that binds Galectin-9 and negatively regulates T cell function [97,100]. The first phase 1b trial to evaluate the efficacy of TIM-3 blockade and dual TIM-3 and PD-1 blockade in AML/MDS (NCT03066648) is underway.

Understandably, blocking a single checkpoint inhibitor is not enough. Indeed, many ongoing trials focused on combinatory therapies (NCT03066648, NCT02530463, NCT01757639).

## 11. Bispecific Antibodies

Blockade of several targets might result in better efficacy; hence the ability of bispecific antibodies (BsAb), tri-specific or multipotent antibodies that can engage with multiple epitopes represents a promising platform to enhance therapeutic efficacy. For example, bi-specific T cell engagers (BiTE) consist of two scFvs, and one engages with T cells. Simultaneously, the other is directed against the tumor antigen such as CD33, thereby engaging endogenous T cells with CD33^+^ AML blast for T-cell-mediated killing. Many different BiTE have shown their efficacy in preclinical studies in AML [101,102,103]. The suitable target for the BiTE is an antigen highly expressed on AML cells but nearly absent on normal cells. Most of the BiTE used in clinical trials against AML targets CD123, or CD33 [104]. There are several early-stage trials using BiTEs (NCT02152956; NCT02715011; NCT02730312; NCT03214666; NCT02520427; NCT03224819; NCT03144245; NCT03038230). Targeting of CD33 by AMG330 and AMV564 BiTE (NCT02520427 and NCT03144245) will also affect MDSC. Whether their deletion will favor AML cure is still unclear, as their levels in BM could either be associated to MRD or relapse following chemotherapy induction [105,106]. The ongoing clinical study results will elucidate how safe and effective this approach is to treat AML. Nevertheless, a standard limitation of all T-cell redirecting therapy, including BiTE, is the cytokine release syndrome (CRS) [107]. Importantly, systemic T cell dysfunction and lack of broad AML target antigen remain a challenge for BiTE based therapies.

## 12. Adoptive Cell Transfer

### 12.1. Hematopoietic Stem Cell Transplantation (HSCT)

Allogeneic HSCT treatment is the transplantation of multipotent hematopoietic stem cells from an HLA-compatible donor. After remission, allo-HSCT shows excellent results in young patients [108] due to the particularly potent immunotherapeutic efficacy of the allogeneic graft-versus-leukemia (GVL) effect; unfortunately, in elderly patients, the effectiveness is less clear [109]. Multiple approaches are under investigation to improve the after-transplantation overall survival [109] in elderly patients. They range from the combinations with different chemotherapies [110] and radiotherapies to enhance T cells’ metabolic activity restoring the GVL [111].

### 12.2. Adoptive T Cells

The direct use of adoptive T-cell involving expansion of autologous T cells has shown great potential in mediating durable and complete remission in chemo-refractory cancer patients. Development of CAR-T cells are T cells equipped with a Chimeric Antigen Receptor. Chimeric receptors are composed of an extracellular domain that binds the target antigen (mainly an ScFv from an antibody) and an intracellular domain consisting of the TCR signaling (CD3z) and different combinations of costimulatory features. Equipping T cells with a CAR makes every T cell active against the tumor.

Nevertheless, more than 25 active clinical trials use this technology to treat AML [112,113]. CLL-1, CD123 and CD33 are the most used targets [114]. CD33 and CD123 are the most promising targets as their expression is ubiquitous in AML blast. Several case reports and pilot studies report the use of CAR T cells in AML [115,116,117]. Unfortunately, CD33 and CD123 are also expressed on healthy HSCs/progenitors and the CAR efficacy is limited by TME [118]. In the effort to avoid severe myeloablation, recently a trial used mRNA-based CAR-T cells. The transient expressions of the CAR do not lead to any effects on the treated patients [115]. A possible approach to this issue could be the co-expression of inducible caspase 9 (iCasp9) in T cells. This construct fuses the intracellular domain of caspase 9, a known pro-apoptotic protein, to a drug-binding domain from FK506- binding protein. Administration of a synthetic molecule drug (AP1903) leads to rapid ablation of T cells [119,120], however at the moment there are no CAR-T trials in AML using this technology. CAR-T in AML is less efficacious than its ability to mediate durable response in B-cell leukemia and lymphomas [114]. Efforts are ongoing to boost the CAR effectiveness in AML. New-generation CARs rely on gene encoding addiction for a cytokine to activate innate immunity and sustain the CAR action [121,122,123,124]. Many different strategies have been developed to diminishing the risk of either “on-target, off-tumor” toxicities or off-target recognition. Roybal et al., show how the synthetic notch receptor allows the CAR expression only after recognizing another antigen [125]. Others linked the activation to the simultaneous recognition of both the antigens [126]. Bi-specific CARs, in which the ectodomains are composed by two different ScFv capable of recognizing two different antigens [127,128,129] are promising candidates to target dual antigens on AML blast, hence, increasing specificity and efficacy. The AML microenvironment seems to be one of the main obstacles to overcome to bring CAR-T cells to success in AML settings [130]. The “commuting CAR” are chimeric receptors able to transform an inhibitory signal into an activating one. It is not surprising that a CAR able to bind TGFβ showed to be effective in modulating the TME [131,132]. CAR Switch receptor can mitigate the immune-checkpoint function. CTLA4:CD28 and PD1:CD28 CAR can increase CAR-T cell effectiveness [133,134]. The addiction of the CD28 intracellular domain transforms the immune-checkpoint into a costimulatory molecule, able to sustain the CAR-T action.

### 12.3. Adoptive NK Cells

Natural killer (NK) cells are innate lymphoid cells specialized in controlling tumor growth and metastasis. NK cells do not require pre-stimulation to perform their effector function, resulting from a balance between activating and inhibitory signals received from cell surface receptors. Tumor cells express molecules that either stimulate or suppress NK cells’ responses by engaging their relative activating or inhibitory receptors [135]. Tumor cells that down-regulate the expression of activating receptors ligands off their surface escape the immune surveillance and, for that matter, cytotoxicity by NK cells [136]. NK cells might recognize the missing expression of the MHC when they encounter mismatched allogeneic cells, referred to as missing self-recognition [136].

NK cells are activated against killer immunoglobulin-like receptor (KIR) ligand-mismatched cells. AML is susceptible to alloreactive NK cells in vitro [137]. Moreover, in haploidentical transplants on AML patients, donor versus recipient NK cell alloreactivity reduced relapse risk [138]. Various approaches have been attempted: short term activated or longer term expanded NK cells, from sources including apheresis product, HSCs differentiated NK to irradiated NK cell lines. From the early attempt in 2005 [139] it was clear that the TME and the persistence of the NK cells is a priority to have a robust response, as demonstrated by the depletion of Tregs by the IL-2 diptheria toxin protein [140]. Multiple clinical studies tried different ways to systemically administrate IL2 or IL-15 in order to improve the NK cells function and persistence [141,142,143,144,145]. Although the results were promising, the effects were not enough to achieve complete remission in the majority of the patients. Even the NK-92 cell line was used in a phase I trial as it appeared to be a safe and off-the-shelf approach. But the low persistence and the limited response led to a stop of the program [146]. One promising attempt was the use of cord blood derived NK cells, but in the absence of cytokine administration, low persistence and a poor level of chimerism was stated [147]. Although NK cells offer a suitable platform for cell immunotherapies in AML and an alternative to T cells in CAR therapies [148], CAR-NK cells have only recently been used in an AML setting [149,150]. Unfortunately, the pool of available targets for a CAR remains limited in AML and can lead to NK cells fratricide. CD33 expression was stated in one subset of activated NK cells [151,152]. Several reports assessed the in-vitro function of CD123 CAR in NK cells, but failed to show a robust response in mouse models [149,153,154,155]. NKG2D CAR enhances NK cell activity and are not subject to downregulation encountered with endogenous NKG2D in AML [156]. NK-92 and primary CAR-NK cells equipped with NKG2D CAR have been evaluated pre-clinically. Moreover, activated NKG2D-CAR-NK cells appeared to outperform CAR-T cells in one animal model [157,158]. This leads to the recently opened phase I clinical trial of a haploidentical donor derived CAR-NK product targeting NKG2DL in MDS and AML (NCT04623944).

CAR-NK cell therapies in AML are still in an early stage of development, but promising results and new approaches of the forementioned issues can greatly improve the effects in the near future.

## 13. Conclusions

Although T-cell quantitative and qualitative deficiencies characterize AML, they have been reversible and restored through treatment. However, the actual treatments (chemotherapy, allo-HSCT) remain largely unchanged over the past decade and lead to high mortality rates. Immunotherapy now represents a highly promising AML cure approach, notably for the elderly. The increasing knowledge of the mechanisms leading to anti-leukemic T-cell response escape (thymic dysfunction, exhaustion, regulatory T cells involvement) will delineate how best to integrate these therapies for optimal patient survival (time considerations, combinations…). A plethora of clinical trials is ongoing to test these different strategies and success will surely be achieved.

## Data Availability

Data sharing is not applicable as no new data were created or analyzed in this article.
